# Developing Public Service Announcements to Help Prevent Suicide among Young People

**DOI:** 10.3390/ijerph18084158

**Published:** 2021-04-14

**Authors:** Maria Ftanou, Nicola Reavley, Jo Robinson, Matthew J. Spittal, Jane Pirkis

**Affiliations:** 1 Centre for Mental Health, Melbourne School of Population and Global Health, The University of Melbourne, Melbourne, VIC 3053, Australia; nreavley@unimelb.edu.au (N.R.); m.spittal@unimelb.edu.au (M.J.S.); j.pirkis@unimelb.edu.au (J.P.); 2 Orygen, Parkville, VIC 3052, Australia; jo.robinson@orygen.org.au; 3 Centre for Youth Mental Health, The University of Melbourne, Parkville, VIC 3052, Australia

**Keywords:** suicide prevention, public service announcements, young people

## Abstract

Background: Suicide is the leading cause of death among young people in Australia. Media campaigns have the potential to reach a broad audience, change attitudes and behaviours, and, ultimately, help prevent suicide. Little is known about the type of content or format suicide prevention media message should take to help prevent suicide among young people. Objective: the objective of this study was to involve young people aged 18 to 24 years in developing three suicide prevention public service announcement (PSAs) targeting young people at risk of suicide appropriate for testing in a randomised controlled trial (RCT). Method: fifteen young people attended at least one of four workshops in Melbourne, Australia. The workshops focused on exploring the appropriateness of three key suicide prevention media PSAs: “Talk to someone”, “Find what works for you”, and “Life can get better”. Young people also provided input into message content, format, and design. Results: participants perceived that all three suicide prevention PSAs were useful and helpful. Participants were concerned that the PSAs may not be suitable for nonwestern cultural groups, could trivialise psychological suffering, and that the actions they promoted could seem distant or unattainable to young people at risk. The featuring of young people, especially young people with hopeful narratives of how they overcame a suicidal crisis, was considered to be an important characteristic of suicide prevention PSAs targeting young people. Conclusions: Developing suicide prevention PSAs with young people is rare but essential to better understand young people’s needs and improve the quality of suicide prevention media PSAs. Further research is needed to evaluate the impact of suicide prevention PSAs developed by young people, for young people.

## 1. Introduction

Suicide is the leading cause of death among young people in Australia. In 2019, 3317 people died by suicide and 480 of these were young people under the age of 25 [1]. Young adulthood is a particularly challenging time, characterised by increased self-focus, independence, identity exploration and transition [2]. By the age of 24, 75% of mental health problems, such as depression, anxiety, and substance abuse will have been diagnosed [3,4], and young people’s reluctance to access support may exacerbate isolation, helplessness and risk of suicide.

Suicide usually results from a unique and complex interaction between a number of risk factors [5,6]. These risk factors can be clinical (e.g., history of mental illness, prior suicide attempts) [7,8,9]; personality and cognitive-based (e.g., impulsivity, hopelessness, poor coping styles) [10,11]; environmental/situational (e.g., history of abuse or trauma, access to means, limited access to help) [10,12,13,14]; genetic [15,16]; and neurobiological (e.g., hyperactivity of the hypothalamic–pituitary–adrenal axis stress response system, impairment of the serotonin neurotransmitter system) [17,18,19,20]. 

Globally significant efforts and investments have gone into research and service development to help prevent suicide among young people. There is, however, a lack of knowledge about which interventions are effective and how to best go about combatting youth suicide [21,22]. As young people are increasingly connected to a variety of platforms, media campaigns may offer an opportunity to engage with young people, educate them about suicide, and help them change situations, feelings, thoughts and behaviours that lead to suicide [23,24]. Most media campaigns have public service announcements (PSAs), with key messages at their core.

There have been a handful of studies across age ranges that have evaluated the impact of suicide prevention media campaigns [23,24,25,26]. The results of these studies have been mixed. Some studies indicate that media campaigns have positive impacts, such as improved knowledge and awareness, attitudes and help-seeking intentions and behaviours. Others have shown that campaigns have no impact on attitudes, help-seeking intentions or behaviours. Conversely, some have even been associated with negative impacts, such as a decrease in help-seeking intentions [23,24,25,26,27,28]. 

Very few studies have explored the precise type of media content that may have a preventative effect. A total of 21 experts (professionals with knowledge about suicide prevention or media campaigns and people with a lived experience of suicide) attending an Australian workshop aimed at developing suicide prevention media messages generally agreed that suicide prevention messages should include two characteristics: (1) validating the struggles experienced by a person at risk of suicide; and (2) promoting help-seeking behaviours [29]. Nicholas, Rossetto, Jorm, Pirkis, and Reavley (2018) conducted a Delphi study that examined the type of messaging suicide prevention experts and people with lived experienced of suicide perceived to be useful. They found that there was general agreement between suicide prevention experts and people with lived experience and that messages that encouraged directly asking people at risk about their suicidal intent, listening without judgment and expressing care were most helpful [30]. Although these types of messages appear to be helpful, it is unclear whether young people will relate to, or engage with, such messaging. 

Theories of behaviour change can provide a conceptual explanation of the relationship between suicide prevention and the media [31,32,33,34,35]. These theories suggest that a behaviour such as help-seeking for suicide can be learned through observational modelling, repetition and reinforcement. They posit that an observer is more likely to imitate or carry out a behaviour if they are somehow able to relate or identify with the person performing the behaviour, or that the behaviour is perceived as acceptable by their peers [31,32]. Therefore, involving young people in the development of suicide prevention PSAs may help craft suicide prevention messages that are safe, effective, and resonate with young people. Only two studies have incorporated the perspectives of young people into the development of suicide prevention media messages, and both these studies utilised high school students in the development of suicide prevention messages [36,37]. 

The objective of this study was to safely involve young people aged 18 to 24 years in developing three suicide prevention PSAs that were appropriate for testing in a subsequent randomised controlled trial (RCT). 

## 2. Method

This study was approved by the Health Sciences Human Ethics Sub-Committee at the University of Melbourne (Ethics Approval Number: 1341188).

### 2.1. Study Design 

A participatory methodological approach was adopted [38,39]. Participatory approaches are person-centred and give a voice to the consumers of products and interventions. The level of consumer involvement can vary from consumers being information providers to them being co-designers, innovators and evaluators. Consumers in this study were primarily involved as information providers and contributors. This approach was deemed suitable, as only very rarely have young people been included in the development of suicide prevention interventions. A series of workshops were conducted to gain insight into what young people perceived to be important characteristics of suicide prevention PSAs. A workshop format was chosen because they provide participants with an opportunity to interact with one another, build on each other’s ideas, provide constructive feedback to facilitators, and allow for the co-development of products. The workshop format also provided facilitators with the opportunity to observe participants and, in this case, ensure their safety throughout the project [40,41]. 

### 2.2. Developing the PSAs

Three suicide prevention PSAs were developed through consultations with suicide prevention and youth mental health experts and four workshops with young people.

#### 2.2.1. Consultations with Suicide Prevention and Youth Mental Health Experts

Five leading Australian researchers and clinicians in the field of suicide prevention and youth mental health (some of whom were members of the research team) were emailed by the research coordinator (MF) and asked to draw on their expertise and knowledge and write down key themes or media messages that they thought would be safe and potentially effective to include in a suicide prevention campaign targeting young people. They were asked to email these to the research coordinator.

The research team met and reviewed the ideas with String Theory Creative, an independent media and digital communication agency. The research team agreed that the three ideas detailed on Table 1 were well supported by evidence and theories of behaviour change, and were safe to present to young people to develop. All three of these ideas targeted young people at risk of suicide.

An additional three PSA ideas, targeting family and community members who might be concerned about someone at risk of suicide, were also considered; the findings in relation to these are not discussed here. 

#### 2.2.2. Workshops with Young People 

Four workshops were conducted at the University of Melbourne to explore both the content and format of suicide prevention PSAs that young people perceived would be effective in preventing youth suicide. Three group facilitators (MF, String Theory Creative) with expertise in media and suicide prevention conducted each of the workshops. Two of the facilitators identified as female and the other as male. MF is a research fellow and clinical psychologist. No prior relationship between the participants and researchers existed prior to the commencement of the study. The first two workshops were approximately 90 minutes in duration and considered message content and format suitable for suicide prevention PSAs. Feedback from participants about the content and format of the three PSAs was then developed by String Theory Creative into the three suicide prevention PSAs.

The second two workshops provided participants an opportunity to review the audio and visuals of the developed PSAs and to provide further feedback on the final PSAs’ content and format. Participants who were not able to attend one of the second workshops were provided with opportunity to meet individually with the research coordinator to review the final PSAs.

At the end of each workshop or meeting with the research coordinator, all participants were provided with crisis helpline numbers. They were also each given an AUD 25 movie voucher in recognition of their contribution. 

### 2.3. Participants

Young people aged 18 to 24, with knowledge of mental health issues, suicide and its prevention, or media campaigns, were actively recruited via word of mouth and through youth mental health advocacy organisations and university networks. These participants were specifically sought out because it was perceived that they would have a good understanding of mental health issues experienced by young people, as well as the strategies or coping styles that young people use to overcome such difficulties. The researcher coordinator (MF) contacted key people at the following organisations (some of which were members of the research team) and informed them of the study: Orygen (a youth mental health specialist service); the Centre for Mental Health at the University of Melbourne; and String Theory Creative. Over a two-week period, these individuals approached potential participants and informed them of the project. Interested participants were either referred to the research coordinator or gave permission to be contacted by the research co-coordinator (MF) directly by phone. All potential participants were provided with an over-the-phone explanation of the nature and purpose of the study. If they agreed to take part, they were invited to attend one of the two initial workshops where they were given a verbal and written explanation of the study and were asked to sign a consent form. Exclusion criteria included young people not proficient in English or who had current suicidal thought or plans. All participants that were referred to the study chose and consented to take part.

### 2.4. Workshop Materials

#### 2.4.1. Participant Characteristics Questionnaire

Participants completed an eight-item sociodemographic questionnaire comprising items about gender, education, employment, relationship status, past mental health problems and living arrangements.

#### 2.4.2. Preliminary PSAs

Participants were provided with a hard copy of the messages outlined in Table 1 to read.

#### 2.4.3. Facilitator’s Discussion Guide

A discussion guide was developed to elicit participants’ views about the types of suicide prevention messages they considered to be engaging, useful and encourage positive actions. In the first two workshops, participants were asked to (1) review the appropriateness of the messages; (2) discuss any potential positive and negative consequences of each PSA; (3) consider the types of creative concepts or approaches that might be most engaging and appealing to young people; and (4) explore the best way to deliver the message for maximum impact. 

At the second two workshops, participants had the opportunity to further review the final content of the message, listen to the audio and provide feedback on the visual of the PSAs.

#### 2.4.4. Workshop Safety Procedures

All young people who attended the workshops were provided with the details of crisis helplines to contact, should they require additional support. Participants were also informed that if they experienced distress throughout the workshop that they could take a break and seek support from the research coordinator (MF). At the end of the workshops, participants were again encouraged to contact the research coordinator (MF) to discuss any issues raised by the study.

### 2.5. Data Collection and Analysis

Facilitators took notes during the workshops and, at the end of each workshop, key points were summarised. The first two workshops were also audio recorded and transcribed. The transcription was not provided to participants. Results were analysed using a thematic analysis approach that was similar to those used in Braun et al. (2020). Notes and transcripts were read and re-read by the research coordinator (MF) and statements and information relating to media message content or format were highlighted and grouped into themes. The main themes that emerged were discussed with the research authors and other workshop facilitators.

The second two workshops, although audio-recorded, were not transcribed. Notes were taken during the workshop by facilitators and changes to the PSA messages were made during the session with participants present. 

## 3. Results

Table 2 provides an overview of the participants’ characteristics. In total, 15 young people (nine females and six males) attended the initial two workshops, and nine participated in the subsequent workshops. The majority of participants were aged between 18 and 20 years. Most of the participants were tertiary educated (67%), employed (87%), resided with family (73%), were born in Australia (93%), and spoke English as their main language (93%). A third of the young people had a prior history of mental health problems and had received treatment.

### 3.1. PSA Message Content

#### 3.1.1. “Talk to Someone” PSA

Workshop participants discussed both the appropriateness and potential consequences of the “Talk to someone” message. Participants were generally positive about this message. They perceived this message to be particularly relevant as young people are often “*ashamed*” or “*afraid*” to reach out to talk about suicide. One participant described their own difficulties stating: “*I’ve always found the hardest thing is to be able to say I’m not doing that well at the moment, even if it’s on any level to someone, because you always have that sort of stigma that you know everyone will talk about physical diseases but mental is a bit more like taboo to talk about*”. Several participants believed that giving people permission to talk about suicide might lead to increased help-seeking and normalising of difficult emotions. 

Some workshop participants also acknowledged that encouraging young people to talk about suicide could help debunk myths and misconceptions such as “*young people who talk about suicide are attention seeking*” *or that* “*talking about suicide can cause suicide*” and reduce the overall stigma around mental health problems and suicide. 

Many participants reported that this message would be appropriate to both people at risk of suicide and their peers, friends, and family members. One participant indicated that such messaging informs both “*families and the general public that if a young person speaks out about suicide that they need to listen.*”

Several participants expressed concerns with this message. Some participants were worried that the message “Talk to someone” was “*too broad*”, “*too common*” “*too cliché*” or “*too impersonal*”, did not acknowledge the difficulties associated with talking about suicide, and that divulging too much might have longer term consequences. These participants recommended that the message could be improved by including information and strategies on “*how*” or “*who*” to talk to about suicide.

Some participants also indicated that young people from minority cultural groups might not relate to the “Talk to someone” message, especially if discussing suicide or mental health problems was not encouraged in their culture. These participants were worried that such messaging could result in young people feeling rejected or marginalised, potentially exacerbating their suicide risk. A few participants were also concerned that if young people talk about suicide with other young people in similar situation, suicide could be encouraged, and that young people might not get the help that they need. These concerns are exemplified by the following remarks:

“*We live in a multicultural society, and some cultures don’t find it easy to share with each other in their family. You couldn’t really [share], if no one’s really going to be talking about it.*”

“*I think there’s a bit of a subculture, especially among teenagers, of suicide being a popular thing and being a thing that’s discussed quite often … if they’re encouraged to talk about it with their friends who encourage suicide or encourage that sort of thinking, [this] may not be that healthy.*”

To mitigate these concerns, participants recommended having people from different cultures appear in the suicide prevention PSAs, or that alternative PSAs be developed to target specific high-risk cultural groups such as Aboriginal and Torres Strait Islander young people. They also suggested that PSAs discuss or list a variety of people and professional support organisations that young people can reach out to for help. Helping young people find ways to reach out and talk about suicide as well as providing them with appropriate sources of support was thought to empower young people to self-manage suicidal thoughts.

#### 3.1.2. “Find What Works for You” PSA

Workshop participants considered the potential benefits of the message “Find what works for you”. They particularly appreciated the direct nature and structure of this message. Some participants indicated that offering young people concrete ideas and alternative options and encouraging those at risk to set small and achievable goals could help provide them with a sense of purpose and improve their self-efficacy and confidence. Participants made comments such as the following:

“I think that when you’re thinking about suicide it’s because you don’t feel like you have any options left, and this ad is kind of offering things you can do. It gives you more options.”

“If you do have to go back to bed and sleep for 19 hours, the next day you can try again, and it’s all about the little steps that are achievable.”

Some participants also raised concerns about this message. Specifically, they were worried that at risk young people may lack the motivation to engage with the strategies or that if they tried the suggested options and were still experiencing suicidal thoughts, they might feel the only option remaining was to harm themselves. A few participants were worried that the PSA was impersonal and did not highlight how hard it might be to help oneself. The following comments highlight some of the participants’ concerns:

“You could go through that checklist and make sure that you’ve done 10 of those things for a whole week and you know what you might just reach a breaking point and end up killing yourself anyway, and… you’ve avoided getting real help because you think that this checklist that a TV commercial has told you [was] foolproof.”

“A little bit too sort of like non-personal, so I sort of want to think of it like a survival checklist. To me it seems sort of like it’s very structured and you do this, this and this, and it doesn’t change no matter who it is, and I think there’s sort of a situation for every person that is going to be slightly different.”

To overcome some of these concerns, participants indicated that the message could include information about both formal and informal supports, and recommended behaviours that could be achievable, even for someone who is at a very low point and finding it difficult to function. One participant also suggested adding a disclaimer that these behaviours: “*might not work for you but that other options exist*”.

#### 3.1.3. “Life Can Get Better” PSA

Workshop participants also discussed the positive and negative consequences of the message “Life can get better”. In terms of positive impacts, some participants particularly valued this message because it provided hope and highlighted that situations and feelings change and “*won’t last forever*”. One participant noted that having people work through suicidal feelings could help motivate other young people to work through their own personal circumstances. This participant said:

“If I was at a very suicidal point and I see someone else who was at that same sort of point as me but was talking about their life and talking about where they’re going that would give me motivation to not want to do it [end my life].”

On the other hand, some participants were worried that this message may be perceived as remote, unrealistic, or unbelievable. One participant said:

“It’s so distant that idea of things will be better, and there’s a lot of steps that you have to take to get there, and it’s almost like that idea of there being so much work, and maybe they won’t get to that point, so I don’t actually think that it’s significantly helpful in that situation.”

Some participants were concerned that the message potentially minimised young people’s hardship and failed to acknowledge the struggle that those with suicidal thoughts go through. To overcome this shortcoming, one participant suggested depicting the struggle that the person at risk experiences. They said:

“You can show that people are really upset, they were really down, but then they came out of it. You need to show the struggle.”

### 3.2. Creative Concepts for Suicide Prevention PSAs

Workshop participants were asked to consider the types of creative concepts or approaches that might be most engaging and appealing to young people. Most participants agreed that suicide prevention PSAs targeting young people should be delivered by young people or feature young people “*who [have] considered suicide in the past and have come out of that dark place*”. Participants thought there should be stories of hope that describe the journey a person at risk of suicide went through to overcome their suicide-related thoughts and behaviours. One participant said:

“It’s really difficult for someone who’s in a dark stage to kind of see that light at the end of the tunnel, because they can’t identify with that, but they can identify with someone who’s in that same spot. So I think you’d have to be really careful how you did that, because you’d have to show them yes they were exactly like you are, they were really upset, they were really down, but then they came out of it.”

For the suicide prevention media message to be relatable, participants suggested featuring young people engaging in everyday activities that included places, music and social situations that are familiar and accessible to the majority of young people. The majority of participants discouraged the inclusion of rates or suicide statistics as they thought that would be perceived as technical and may even prevent young people accessing help. They made comments like:

“I’m always very kind of sketchy about statistics, because I feel like people just throw them out there, as persuasive technique.”

“Statistics don’t really work for me, but I know lots of people who are more mathematically minded would definitely benefit from hearing statistics, and maybe hearing like one in ten people are suffering this that might help a lot of people. Other might think well if other people are going through this then I have no reason to ask for help, because you know there are other people, I guess, who are worse off.”

Young people believed that PSAs needed to be inclusive and relatable to a broad range of young people from diverse cultural backgrounds, as well as different ages and genders. Participants particularly appreciated vox-pops because they could easily capture the opinions and experiences of a number of different young people. One participant said: “*I like the vox-pop idea*, *you can get 10 separate stories*”. 

In terms of emotional tone, participants tended to discourage the development of PSAs that were humorous or evoked fearful response from viewers. Participants said things such as “*suicide is not humorous*” or PSAs “*should not be doom and gloom*”. Participants suggested that suicide prevention PSAs which adopted a serious, positive and hopeful tone were more likely to engage young people and help create change.

### 3.3. Modes of Delivering the Suicide Prevention PSAs

Workshop participants were asked to consider the best avenues to deliver the suicide prevention PSAs for maximum impact. They suggested that PSAs should be featured across multiple platforms, including television, cinemas, and social media. Participants agreed that social media had a broader reach but indicated that messaging could be reinforced through more traditional media or through relevant youth related venues (e.g., by universities, health professionals’ services circulating pamphlets with the main campaign message and associated strategies). They made comments such as the following:

“I think you could have a really general kind of campaign like commercial kind of thing, but then maybe follow it up with a website or a link that they could go to with that follow up information, so start the conversation about suicide, that kind of message, and then for more information follow this website.”

“Yeah like I can imagine if this was in a pamphlet say at a school where someone’s gone for help and they’ve got a tool to give them alongside scheduling regular appointments.”

### 3.4. Safety of Workshops

None of the participants that attended the workshops reported feeling distressed or requested additional help or support from the research team or workshop facilitators.

### 3.5. Final PSAs

The findings from the workshops resulted in String Theory Creative producing the three suicide prevention PSAs. The final suicide prevention PSAs, as suggested by the workshop participants, adopted a vox-pop approach. A number of young people from diverse backgrounds were featured in each media message, undertaking everyday common activities (e.g., waiting at a tram stop, walking along a beach, spending time in their bedrooms). The final content of each PSA is presented in Table 3. All three PSAs encouraged connectedness, self-efficacy, and finding alternative ways to manage suicidal thoughts. 

## 4. Discussion

### 4.1. Summary of Findings

Our study was the first to involve young people aged 18 to 24 years in the development of suicide prevention PSAs. It provided insights into the types of PSAs that young people perceive to be effective in combating youth suicide. Our findings also highlighted that young people recommend the adoption of media messages that provide suggestions on how to get help, how to talk about suicide, and strategies that could help overcome suicidal thinking. Such messaging was thought to improve self-efficacy. Research on the media coverage of suicide has also found that newspaper articles or films that feature a main character positively coping or using alternative strategies to help with suicidal thoughts is associated with improved life satisfaction [42,43]. The young people in our study were also concerned that, if messages were not crafted carefully, they could lead to decreased help-seeking and isolation. In particular, they were worried that the messages “Talk about it” may not resonate with young people from culturally diverse groups and that both the “Find what works for you” and “Life can get better” messages might be perceived as trivialising psychological suffering, or that suggested actions could seem too distant or unattainable for young people at risk. These concerns should not be minimised, as highlighted in an RCT involving 426 adolescents, where Klimes-Dougan et al. (2009) demonstrated that the PSA “Prevent suicide: treat depression” had unintended consequences (e.g., reduced help-seeking intentions) for a specific subgroup of high-risk young people.

Similar to the findings of Braun et al. (2020), young people in our study flagged the importance of featuring young people in suicide prevention PSAs. Featuring young people was thought to help create more personal, authentic and relatable PSAs, compared to those that feature technical information such as suicide statistics. Lastly, for suicide prevention PSAs to have a broad reach, young people emphasised that messages should be delivered via multiple platforms including television, cinemas and social media.

These findings highlighted that young people can be safely engaged in the development of suicide prevention PSAs and that their contribution could be valuable. Their involvement in this study lead to an understanding of the types of PSAs that young people thought would be useful in campaigns, which supported the development of three suicide prevention PSA for testing in a RCT [44].

### 4.2. Co-Designing PSAs with Young People

Given that young people have not previously been widely involved in the development of suicide prevention PSAs, and to minimise any potential harm, we took a very cautious approach in their engagement in the process. Due to the lack of evidence on the types of suicide prevention PSAs that are safe and effective, this study did not involve young people in the initial brainstorming of message content for the PSAs. Rather, suicide prevention and mental health experts developed messages for the initial PSAs to ensure that they were evidence-based, consistent with media reporting guidelines for suicide, and were underpinned by behaviour change theories. This process of having professionals set the initial themes of the PSAs may not have completely captured issues in suicide prevention that are meaningful or relevant for young people. When young people were subsequently engaged in the content through workshops, professionals were not included as participants to ensure that the young people felt comfortable and empowered to express their views, actively contribute, and to limit any socially desirable answers. It is possible that that combined workshops with media/suicide prevention experts and young people could have improved collaboration, idea generation and knowledge exchange.

More active engagement of young people in the development of suicide prevention PSAs through a co-design process may address these shortfalls and ensure that PSAs address the needs of those that they target. Co-design is a process by which experts such as researchers, clinicians and designers come together with consumers to develop, implement, and evaluate interventions, services, and products [45,46,47]. Co-design methodologies have two core features: (1) consumer expertise is given equal value to professional expertise (i.e., that lived experience is as important as scientific knowledge); and (2) consumers have equal responsibility and decision-making powers to other stakeholders [45,46,47,48].

Co-designing PSAs with young people would involve engaging them throughout the whole developmental process, from problem identification, brainstorming ideas and solutions, and designing, to delivering and evaluating the impact of the PSAs. It would go beyond giving information to maintaining active participation. Keeping young people engaged in a co-design process involves setting clear role expectations, being flexible regarding their level of commitment, reimbursing young people appropriately, recognising and valuing their experience and contributions, and ensuring that they are safe and well supported throughout the process [47,48].

In the absence of a strong evidence-base about the type of media content that may have a preventative effect, co-design processes can also be guided by theories, such as the Theory of Planned Behaviour (TPB) [32,34]. The TPB argues that intentions are the best predictor of behaviour and are influenced by three key factors: attitudes, subjective norms, and perceived control. PSAs can be designed to target one of these three components. For example, to modify unhelpful attitudes towards suicide, PSAs can raise awareness of the myths of suicide and benefits of help-seeking. To shift social norms, PSAs can normalise help-seeking by featuring young people role modelling and reaching out to accept help from a variety of sources. To influence perceived behaviour control, PSAs can disseminate information about evidence-based interventions and how to access supports.

In recognition of the fact that co-design might yield more optimal PSAs, we have conducted a subsequent study in which we asked young people aged 18 to 24 years to develop their own suicide prevention PSAs aimed at helping a peer or colleague at risk of suicide. We provided information about suicide prevention and PSA production to participating young people, but they were entirely responsible for the content and format of their own PSAs. We asked an independent group of suicide prevention experts and young people to judge the resultant PSAs. We tested the winning co-designed PSA in an RCT. The young people who viewed the PSAs indicated that the PSA was easy to understand, relevant, and encouraged young people to help a friend who was suicidal (Ftanou et al., in preparation).

### 4.3. Limitations

This study had several limitations. Workshop participants were self-selected and had an interest in mental health or their own experience of mental health issues (those experiencing suicide-related thoughts and behaviours were excluded). The sample size was also small and largely comprised young females. Although the young people in the study wanted to ensure that PSAs were inclusive of different cultural groups and they had raised concerns that some messaging might not be appropriate, most of the participants in the study were Australian born who spoke English at home. Future research should target a broader range of ethnicities and cultures to make certain that PSAs are relevant and appropriate across a range of demographics [49]. The small sample size was deemed suitable to help manage any potential risks; however, as a consequence, the PSAs that were developed may not be acceptable or relevant to some groups of young people. The workshops with young people were facilitated by research team members and media experts, which may have influenced young people to provide socially desirable responses. As many as 40 percent of the young people did not attend a second workshop, which may have limited the feedback received about how to adapt the PSAs and distribute them. Lastly, the study did not evaluate the young people’s experience of taking part in the workshop process. An understanding of their experience throughout the process may have assisted in the engagement and retention of participants. 

## 5. Conclusions

Young people’s perspectives on how to best design suicide prevention PSAs that target them are critical. In the current study, young people helped shape our thinking about PSAs that were ultimately created and tested in an RCT [44]. Involving young people in the development of suicide prevention PSAs is rare but has the potential to lead to a better understanding of their needs and improve the quality of the message and format in which it is delivered. Further research is needed to evaluate the actual impact of suicide prevention PSAs developed by young people, for young people.

## Figures and Tables

**Table 1:** Preliminary suicide prevention PSAs.

	Key Message	Aim	Content Ideas
1	“Talk to someone”	To improve understanding of suicide and encourage young people to talk about suicide and seek help for their suicidal thoughts and feelings.	Provide examples of common misconception about suicide and encourage talking about suicide: If someone talks about suicide, they are not serious If someone wants to end their life, there is no way to stop them Provide examples that encourage talking about suicide Talk to someone you trust who will listen to you without judgment—a friend, family member, doctor, health professional or counsellor Explain how you feel Give offers of help a try
2	“Find what works for you”	To help young people identify things they can do to keep living	Provide examples on how a person at risk might be able to help themselves such as: Work out who can support you Set yourself something to do each day Checklist of thing you can do to cope with the problem
3	“Life can get better”	To help young people understand that things can change—that what they feel now will not last forever.	Provide examples of how a person’s life has changed since experiencing suicidal thoughts, such as: Reflecting on achievements big or small Reflecting on changes in relationships Reflecting on special life milestones Reflection on coping strategies they have learnt

**Table 2:** Characteristics of workshop participants.

Demographics		Workshop Participants (*n* = 15)	
		Freq	%
Age	18 to 20 years	8	53.3%
	21 to 24 years	7	46.7%
Gender	Male	6.0	40.0%
	Female	9.0	60.0%
Highest educational qualification	Secondary Year 12	4.0	26.7%
	TAFE	1.0	6.7%
	Tertiary	10.0	66.7%
Currently employed	Yes	13.0	86.7%
	No	2.0	13.3%
Resides with	Alone	1.0	6.7%
	Family	11.0	73.3%
	Shared house	2	13.3%
	Missing	1.0	6.7%
Main language spoken at home	English	14.0	93.3%
	Missing	1.0	6.7%
Country of birth	Australia	14.0	93.3%
	Missing	1.0	6.7%
Mental health history	Yes	5.0	33.3%
	No	9.0	60.0%
	Missing	1.0	6.7%
Accessed mental health services in the past	Yes	5.0	33.3%
	No	9.0	60.0%
	Missing	1.0	6.7%

**Table 3:** Final suicide prevention media PSA scripts.

PSA 1: Talk to Someone	PSA 2: Find What Works for You	PSA 3: Life Can Get Better
I went through this time where I didn’t want to live anymore I just kept thinking about killing myself. I ended up talking to my best mate about it. I talked to my mum; it was such a relief to finally let her know what was going on. I called Lifeline. I went and saw a counsellor and they made me realise I had nothing to be ashamed of. At first it was easier talking to an online counsellor than face to face. Speaking out about my suicidal thoughts was one of the hardest things I have ever done, but it made a huge difference. Reach out and talk to someone. You can do things to change your suicidal thinking.	I went through this time where I didn’t want to live anymore I just kept thinking about killing myself. I started to write my thoughts down to get them out of my head. I made sure I spent time with someone every day. I gave myself something to do and focus on each day, something that gave me a sense of purpose. I did some exercise every day—whatever I could. Eventually I went to see a counsellor. Find what works for you. You can do things to change your suicidal thinking.	I went through this time where I didn’t want to live anymore. I just kept thinking about killing myself. Its’ been a year since I was suicidal, and things are really different now. I focused on what I could change. There was this realisation that my depression was not permanent. I cope with things differently now. I’ve actually started to have fun again. Life can get better. You can do things to change your suicidal thinking.

## Data Availability

Not applicable.
